# Palliative Radiotherapy for Esophageal and Gastric Cancer: Population-Based Patterns of Utilization and Outcomes in Ontario, Canada

**DOI:** 10.1177/08258597211072946

**Published:** 2022-01-19

**Authors:** Shaila J. Merchant, Weidong Kong, Aamer Mahmud, Christopher M. Booth, Timothy P. Hanna

**Affiliations:** 1 Division of General Surgery and Surgical Oncology, Department of Surgery, Queen's University, Kingston, Ontario, Canada; 2 Cancer Care and Epidemiology, Queen's Cancer Research Institute, Kingston, Ontario, Canada; 3 Division of Radiation Oncology, Department of Oncology, Queen's University, Kingston, Ontario, Canada; 4 Division of Medical Oncology, Department of Oncology, Queen's University, Kingston, Ontario, Canada

**Keywords:** palliative, radiation, esophageal cancer, gastric cancer, outcomes

## Abstract

**Objective:**

Patients with incurable esophageal and gastric cancer may develop local symptoms for which palliative radiotherapy (PRT) may be considered. We sought to evaluate patterns in utilization and outcomes of patients receiving PRT for incurable esophageal and gastric cancer in Ontario, Canada using health administrative data.

**Methods:**

Linked health administrative databases were used to identify patients receiving PRT for incurable esophageal and gastric cancer. Primary outcomes were utilization and delivery of PRT, utilization of endoscopic dilation with or without stent insertion after completion of PRT and survival from 1) date of diagnosis and 2) start of PRT.

**Results:**

We identified 2500 patients who received PRT. Mean age was 70 ± 13 years and the majority (75%, *n* = 1873/2500) were male. Over half of the patients had a diagnosis of gastric cancer (58%, *n* = 1453/2500) and began PRT within 6 months of cancer diagnosis (85%, *n* = 2125/2500). Of the 2500 patients in the cohort, 2174 patients received EBRT with few receiving brachytherapy (*n* = 326) or EBRT and brachytherapy combined (*n* = 88). Over the study period, there was an increase in the number of patients receiving PRT (136 in 2007 to 290 in 2016), as well as in the use of advanced conformal radiotherapy techniques. Only 5% (115/2500) required dilation with or without stent insertion after completion of PRT. Median overall and cancer-specific survival of the cohort was 205 days and 209 days from date of diagnosis and 108 days and 110 days from start of PRT.

**Conclusions:**

PRT is an important treatment for patients with incurable esophageal and gastric cancer who present with local symptoms. Utilization of PRT and advanced EBRT techniques increased over the study period. Few patients require endoscopic dilation with or without stent insertion after completion of PRT suggesting that PRT provides favorable symptom control.

## Introduction

Metastatic disease commonly develops in patients with esophageal and gastric cancer.^1–5^ Some of these patients will present with local symptoms such as pain, dysphagia, obstruction, and bleeding;^6^ palliative radiotherapy (PRT) may be considered to address these symptoms^7–9^ with improvement in quality of life.^10^ A systematic review reported that response rates for bleeding, pain and obstruction in advanced gastric cancer are 74%, 67% and 68%, respectively^9^ while a randomized clinical trial in patients with esophageal cancer reported dysphagia relief in 35% of patients receiving radiotherapy.^8^ Existing literature on PRT in this population is largely comprised of small cases series from single institutions; consequently, the extent to which these radiotherapy treatments are utilized and delivered at a population level is unknown. This type of information may help to inform patient-provider decision-making surrounding selection of PRT to address symptoms in patients with esophageal and gastric cancer.

To address these gaps in knowledge for patients with incurable esophageal and gastric cancer, our objectives were to describe the population-based a) utilization and delivery of PRT, b) utilization of endoscopic dilation with or without stent insertion after completion of PRT, and c) survival in patients receiving PRT for esophageal and gastric cancer.

## Methods

### Study Design

Retrospective health administrative database study.

### Cohort

**Figure 1.**
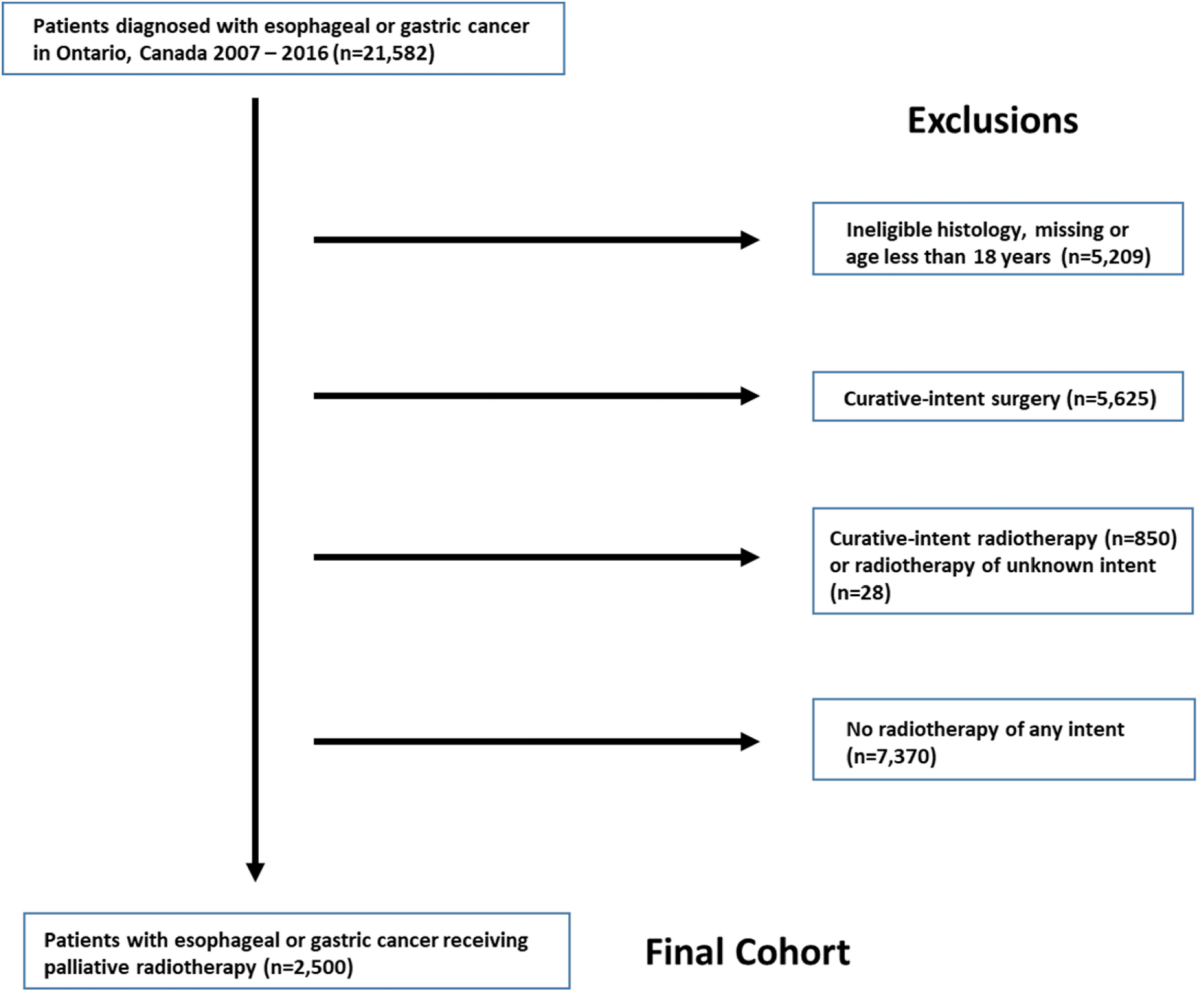
Cohort creation flowchart.

The study population included all patients diagnosed with esophageal and gastric cancer from 2007 to 2016 in the province of Ontario who did not undergo curative-intent surgery or radiotherapy. Ontario has a population of ∼15 million people and a single-payer universal health insurance program. The cohort was identified from the Ontario Cancer Registry (OCR) using the International Classification of Diseases 10th Edition (ICD-10, esophageal C15.X; gastric C16.X). There is no specific ICD-10 code for cancers of the gastroesophageal junction. In patients with multiple gastroesophageal primary cancers (separated by a period of time), the most recent diagnosis was included and for those with two separate gastroesophageal cancers diagnosed on the same day (ie two biopsy results on the same day), the gastric cancer diagnosis was prioritized as a rule. This occurred in a minority of patients. Histologic subtypes such as lymphoma, neuroendocrine tumor, sarcoma and squamous cell carcinoma were excluded. The final list of included histologies is summarized in Appendix Table A1. The initial cohort was then categorized based on receipt of previous surgery and radiotherapy. Patients with ineligible histology or age, a history of curative-intent surgery or radiotherapy, and those who never received radiotherapy were excluded. After these exclusions the final cohort consisted of patients who had received PRT (Figure 1).

### Databases and Linkage

The OCR is a population-based cancer registry that captures diagnostic and demographic information on incident cancer cases in the province of Ontario.^11^ It provides information on vital status and cause of death. The study cohort identified from OCR was then linked, using a unique patient identifier, to information from cancer treatment databases [Cancer Activity Level Reporting (ALR) and New Drug Funding Plan (NDFP) *–*which capture treatment date, radiotherapy dose delivered, number of fractions and intent of radiotherapy treatment. The National Hospital Productivity Improvement Program (NHPIP) codes reported in ALR were used to classify radiotherapy techniques.

Patients undergoing curative-intent surgery were defined as those undergoing excision/resection of the esophagus and/or stomach and with a minimum hospital length of stay (LOS) of 4 days, identified using the Canadian Classification of Health Interventions (CCI) procedural codes updated to 2017 (Appendix Table A2). We specified this minimum hospital LOS to exclude patients who presented for surgical resection but were found to have unresectable or metastatic disease and therefore, did not undergo curative-intent surgery. Endoscopic dilation with or without stent insertion after completion of PRT was identified using CCI codes and included both inpatient and outpatient procedures (Appendix Table A3).

### Covariates and Outcomes

Age and sex were categorized. Patient location was defined as rural or urban based on postal code at time of diagnosis. Socioeconomic status was based on neighborhood income and categorized into quintiles, with quintile 1 representing those areas that were most deprived. Distance to nearest hospital offering radiotherapy services, cancer site (esophageal and gastric), receipt of palliative chemotherapy (systemic chemotherapy or chemotherapy given concurrently with radiation [ie chemoradiation]) and time from initial diagnosis to start of PRT were categorized. Comorbidity information was only available on patients from April 2011 onwards as diagnoses from the preceding five years (up to 2006) were used to calculate the Charlson comorbidity index (*n* = 1644).

We focused on the delivery of external beam radiotherapy (EBRT) as most patients receive this modality in Ontario; a minority of patients receive brachytherapy. PRT delivery was described using dose, number of fractions, and body sites radiated. Radiotherapy techniques were summarized from NHPIP codes and were categorized into 6 levels: advanced radiotherapy (ie stereotactic body radiotherapy [SBRT], Cyberknife, Gammaknife, volumetric-modulated arc therapy, tomotherapy, and intensity modulated radiotherapy), brachytherapy, three or more fields, two field, direct field, and other (ie Electrons, LINAC Extended SSD, LINAC Non-Modulated Technique, orthovoltage and superficial radiation) based on descriptions in the NHPIP codes. Only the first course of PRT was captured and if multiple radiotherapy techniques were delivered in the first course, the following prioritization was utilized to describe technique: advanced radiotherapy>brachytherapy>three or more field> two field> direct field> other.

Specific dates for endoscopic dilation with or without stent insertion were not available in the dataset; therefore, the first hospital admission date after completion of PRT was used as a proxy with a 2-week period for capture of inpatient and outpatient procedures. For multiple events, only the first event was counted.

The primary study outcomes were the utilization and delivery of PRT, utilization of endoscopic dilation with or without stent insertion after completion of PRT and survival from 1) date of diagnosis and 2) start of PRT.

### Statistical Analyses

Characteristics of patients receiving PRT were summarized. Information pertaining to utilization and delivery of PRT and need for endoscopic dilation with or without stent insertion was summarized. Survival from 1) date of diagnosis and 2) start of PRT was determined using the Kaplan-Meier method.

Sensitivity analyses were performed to compare characteristics and survival of patients receiving PRT based on a) palliative intent code in ALR (*n* = 2500) b) dose per fraction ≥300 cGY (*n* = 2511) and c) dose per fraction ≥250 cGy and <850 cGy and ≤15 fractions (*n* = 2573) to ensure that the cohort receiving PRT was accurately identified. These rules for PRT were based on clinical experience and judgment of the radiation oncologists in our group (TH and AM); furthermore, a relevant randomized trial reported that 15 fractions was considered “palliative” treatment in Australia and New Zealand.^8^

Results were considered statistically significant at *p*-value <0.05. All analyses were performed using SAS version 9.4 (SAS Institute, Cary, NC). This study was designed, analyzed, and reported in accordance with the Strengthening the Reporting of Observational Studies in Epidemiology (STROBE)^12^ and Reporting of Studies Conducted using Observational Routinely-collected Health Data (RECORD) statements.^13^

### Ethical Considerations

Ethical approval for the study was obtained from the Human Research Ethics Board at Queen's University, Kingston, Ontario.

## Results

### Study Cohort

During the study period 21,582 patients were diagnosed with esophageal or gastric cancer. Patients ineligible by histology or age (*n* = 5209) and with curative-intent surgery (*n* = 5625) and radiotherapy (*n* = 850) were excluded as were patients without any radiotherapy (*n* = 7370). The remaining 2500 patients received PRT and comprised the study cohort (Figure 1). Characteristics of the cohort are summarized in Table 1. In this cohort, the mean age was 70 ± 13 years and the majority (75%, *n* = 1873/2500) were male. Most patients had a comorbidity index of 0 (56%, *n* = 927/1644) and resided in an urban location (83%, *n* = 2075/2500). Over half of the patients had a diagnosis of gastric cancer (58%, *n* = 1453/2500) and began PRT within 6 months of cancer diagnosis (85%, *n* = 2125/2500). Most patients (63%, 1567/2500) received no chemotherapy after diagnosis.

**Table 1.** Characteristics of Patients Receiving Palliative Radiotherapy (PRT) for Esophageal or Gastric Cancer During the Study Period (2007-2016) (*n* = 2500).

Characteristics	n (%)
**Age**	
≤40	50 (2.0%)
41 to 50	166 (6.6%)
51 to 60	442 (17.7%)
61 to 70	608 (24.3%)
≥71	1234 (49.4%)
mean (SD)	69.5 (13.4)
median (IQR)	70 (60-80)
**Sex**	
Male	1873 (74.9%)
Female	627 (25.1%)
Charlson comorbidity index*	
0	927 (56.4%)
1	385 (23.4%)
≥ 2	332 (20.2%)
**Patient location**	
Rural	413 (16.6%)
Urban	2075 (83.4%)
**Socioeconomic status quintile**	
1 (lowest)	448 (18.0%)
2	572 (23.0%)
3	572 (23.0%)
4	493 (19.8%)
5 (highest)	405 (16.3%)
**Distance to nearest hospital offering radiotherapy services**	
≤ 10 kms	933 (37.5%)
11 to 50 kms	998 (40.1%)
> 50 kms	559 (22.4%)
**Cancer site**	
Esophageal	1047 (41.9%)
Gastric	1453 (58.1%)
**Receipt of chemotherapy**	
Systemic chemotherapy	665 (26.6%)
Chemotherapy concurrent with radiation	268 (10.7%)
No chemotherapy	1567 (62.7%)
**Time from cancer diagnosis to start of palliative radiotherapy**	
3 months	1881 (75.2%)
>3 to 6 months	244 (9.8%)
>6 to 12 months	229 (9.2%)
>12 to 24 months	113 (4.5%)
>24 months	33 (1.3%)

*Performed on subset of cohort for which full comorbidity information available (*n* = 1644).

SD – standard deviation.

IQR – interquartile range.

### Delivery of Palliative Radiotherapy

**Figure 2.**
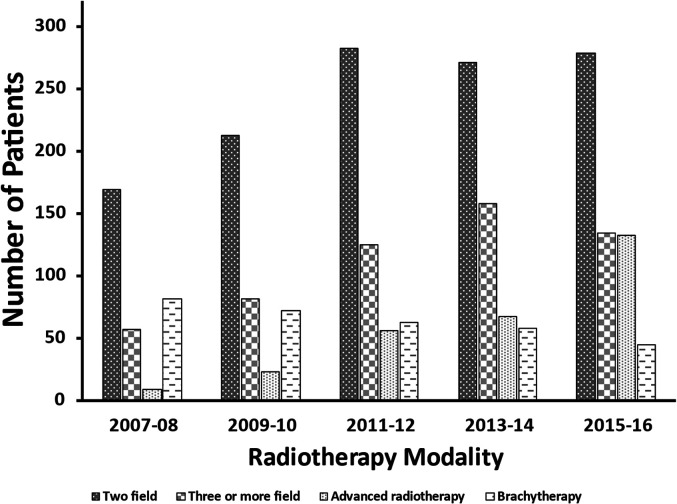
Trends in palliative radiotherapy techniques for all radiated sites by treatment years. Only the first course of palliative radiotherapy was captured and if multiple radiotherapy techniques were delivered in the first course (190 patients), the following prioritization was utilized to capture technique: advanced radiotherapy>brachytherapy>three or more field> two field. Direct field and other technique data not shown due to small cells. Patients from 2006 excluded as no patients received palliative radiotherapy and those from 2017 and 2018 excluded as complete treatment records not available. *Other - Electrons, LINAC Extended SSD, LINAC Non-Modulated Technique, orthovoltage and superficial radiation.

Of the 2500 patients in the cohort, 2174 patients received 2741 courses of EBRT. Few received brachytherapy (*n* = 531 courses in 326 patients) or EBRT and brachytherapy combined (*n* = 176 courses in 88 patients). Subsequent analyses focused on delivery of EBRT only (*n* = 2174). Most patients received 1 course of PRT (83%, *n* = 1812/2174); 13% (293/2174) and 3% (69/2174) received 2 and 3 courses, respectively. The range for number of courses was 1 to 6. The most commonly observed number of fractions delivered were 1 (19%), 5 (37%), and 10 (26%) and the most commonly observed doses were 800 Centigray (cGy) (11%), 2000 cGY (30%) and 3000 cGy (22%). There were no significant differences in commonly administered fractions and doses of PRT for esophagus and gastric cancers (data not shown). The most utilized PRT technique was two field and less commonly three or more field, brachytherapy, and advanced radiotherapy (Figure 2); few patients received direct field or other forms of radiotherapy (data not shown as small cells suppressed). Over the study period, the number of patients receiving advanced radiotherapy techniques increased even when the data was analyzed by site (esophagus or stomach) of irradiation (data not shown).

Over the study period, there was an increase in the number of patients receiving PRT (136 in 2007 to 290 in 2016); increase in numbers of treated patients were also observed when esophageal (65 in 2007 and 123 in 2016) and gastric (71 in 2007 and 167 in 2016) cancers were analyzed separately (Table 2).

**Table 2.** Trends in Receipt of Palliative Radiotherapy by Treatment Year Categorized by Cancer Site. The Table Represents Numbers of Patients Receiving Treatment. Patients from 2006 Excluded as no Patients Received Palliative Radiotherapy and Those from 2017 and 2018 Excluded as Complete Treatment Records not Available.

Year	Esophagus (n)	Stomach (n)	Overall (n)
2007	65	71	136
2008	102	83	185
2009	88	97	185
2010	95	114	209
2011	115	168	283
2012	110	145	255
2013	109	173	282
2014	103	183	286
2015	119	186	305
2016	123	167	290

With respect to number of EBRT courses delivered to body regions, the most treated areas were the esophagus (*n* = 1015/2741), stomach (*n* = 660/2741), abdomen (*n* = 385/2741), spine (*n* = 290/2741) and brain (*n* = 179/2741).

### Utilization of Endoscopic Dilation with or Without Stent Insertion After Palliative Radiotherapy

**Figure 3.**
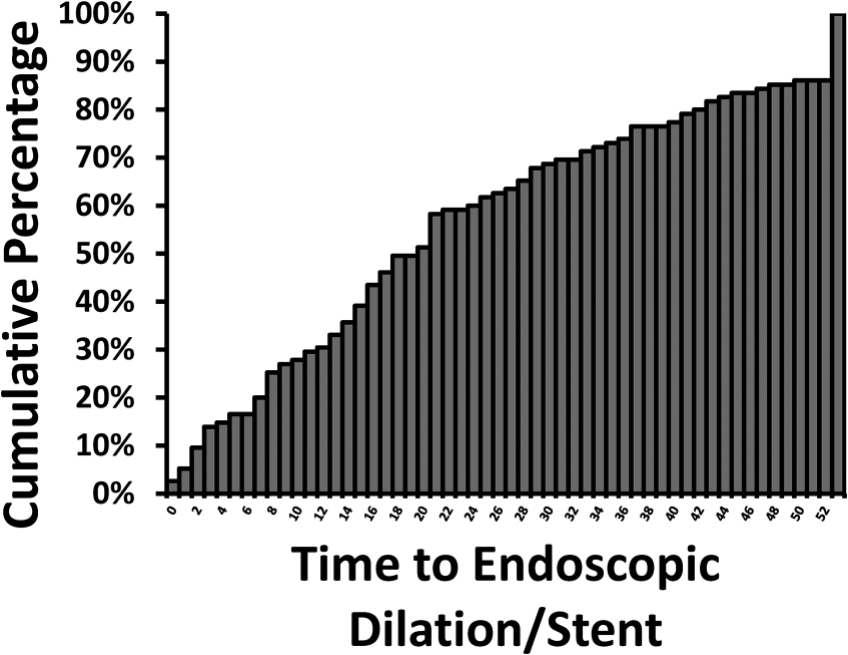
Cumulative incidence of first endoscopic dilation with or without stent insertion after completion of palliative radiotherapy for esophageal or gastric cancer (*n* = 115/2500). If patients received multiple courses of palliative radiotherapy, the intervention was captured only after the last delivered course.

Of patients who received any PRT, 5% (115/2500) required dilation with or without stent insertion after completion of PRT. Most of these patients had esophageal cancer (111/115, 97%). Mean and median time to dilation with or without stent insertion after PRT completion was 196 days and 142 days (range 4, 1268), respectively. Twenty-five percent (29/115) of patients received this intervention within 61 days and 75% (86/115) received it within 263 days of PRT completion (Figure 3).

### Survival of Patients Receiving Palliative Radiotherapy

Median overall and cancer-specific survival of the cohort from diagnosis was 205 days and 209 days, respectively. For patients with esophageal cancer (*n* = 1047), median overall and cancer-specific survival were 198 days and 202 days, respectively. For patients with gastric cancer (*n* = 1453), median overall and cancer-specific survival were 211 days and 212 days, respectively.

Median overall and cancer-specific survival of the cohort from PRT start was 108 days and 110 days, respectively. For patients with esophageal cancer (*n* = 1047), median overall and cancer-specific survival were 115 days and 118 days, respectively. For patients with gastric cancer (*n* = 1453), median overall and cancer-specific survival were 102 days and 104 days, respectively.

For patients with esophageal cancer who received PRT to the primary site (esophagus) versus other body site, median overall survival was 202 versus 178 days, respectively. For patients with gastric cancer who received PRT to the primary site (stomach) versus other body site, median overall survival was 232 versus 204 days, respectively.

Median overall survival was longer in patients with a longer interval between cancer diagnosis and receipt of PRT. Patients receiving PRT from 0-<4 months, 4-<12 months and >12 months from diagnosis had median overall survival 158, 337 and 677 days, respectively.

### Sensitivity Analyses:

Patients receiving PRT according three different coding definitions based on recorded intent, dose and fractionation were compared and this showed no obvious differences in the characteristics and median survival (Appendix Table A4).

## Discussion

In this study we have described the utilization and delivery of PRT in patients with esophageal and gastric cancer in Ontario, Canada. We also report the utilization of endoscopic dilation with or without stent insertion in this cohort which is a clinically meaningful endpoint in routine practice. Most of the patients in our cohort were male and over half had a diagnosis of gastric cancer which is in keeping with commonly reported statistics for gastric and esophageal cancer in Canada.^14^ Most patients received PRT within 6 months of diagnosis and survival was uniformly poor in our cohort and in keeping with published survival data in patients with incurable esophageal and gastric cancer.^14^

EBRT was the most commonly utilized radiation technique in the delivery of PRT and was the focus of this study. Categorization of radiotherapy techniques illustrated that two-field radiotherapy was used most commonly; however, there was also an increase in patients receiving more advanced radiotherapy techniques over the study period. These techniques include SBRT, Cyberknife, Gammaknife, volumetric-modulated arc therapy, tomotherapy, and intensity modulated radiotherapy. The variation in radiotherapy techniques may be related to the body sites radiated; for example, metastatic disease to the brain is more frequently treated with advanced techniques such as stereotactic radiotherapy^15^ and gamma knife.^16^ Our data shows that the most commonly radiated sites of distant metastases from esophageal and gastric cancer include abdomen, spine and brain. Others have shown that in esophageal cancer common sites of distant metastases are liver, distant/non-regional lymph nodes, lung, bone and brain,^17^ similar to our findings. Further research should aim to quantify the added local control and symptom control benefit of more advanced radiotherapy techniques for palliation of esophageal and gastric cancer. In addition, while studies have shown that advanced radiation techniques may minimize toxicity by sparing at-risk organs in patients with lung cancer,^18,19^ similar data is not yet available for patients with esophageal and gastric cancer in the palliative setting, and would be a valuable area of research focus.

We show that there are variations in dose and fractionation of PRT in Ontario, Canada, a finding also reported by others in different parts of the world. A systematic review studied the utilization of PRT for gastric cancer and reported substantial variation in dose and number of fractions;^9^ however, the most commonly observed treatment was 30 Gy in 10 fractions, in keeping with our study. Kim et al. reported that 35 Gy in 14 fractions was the most commonly delivered regimen in patients with advanced gastric cancer.^7^ In a small case series Hiramoto et al.^20^ reported median total dose 42 (range 18-60) Gy and median number of fractions 20 (range 9-30). In a randomized clinical trial Penniment et al.^8^ also reported variation in dose and fraction by geographical region – 35 Gy in 15 fractions was delivered in Australia and New Zealand and 30 Gy in 10 fractions was delivered in Canada and the United Kingdom. These variations in practice may be due to several factors including radiation oncologist training and experience, institutional protocols, body site requiring radiation, and patient preferences and ability to travel for treatment and tolerate toxicity. In Canada, a survey of radiation oncologists treating bone metastases revealed that the most common modality was EBRT with delivery of 20 Gy in 5 fractions; however, there was also variation in practice among the group, including other modalities of delivery such as half body radiation and radionuclides.^21^ A recent study of radiation oncologists reported a variety of behavioral determinants associated with treatment recommendations.^22^ Bradley et al.^23^ reported that, when feasible, patients prefer a single treatment over multiple treatments due to convenience; however, ability to do this depends on the body site requiring treatment. While these data suggest that variation is common, it is less clear whether the variation is associated with differences in patient outcomes. Future research efforts should be dedicated to this.

There is limited literature related to the need for secondary intervention after completion of PRT. Walterbos et al.^24^ reported that a higher dose schedule provided similar symptom relief but was related to a longer time to second intervention (ie re-irradiation or stent placement) compared to a lower dose schedule. In that study, 16% of patients required stent placement after completion of PRT. Murray et al.^25^ reported that 26% of patients treated with palliative EBRT for esophageal cancer required subsequent stent insertion. A randomized trial^8^ reported that esophageal stenting was required in 21% and 31% of patients having completed chemoradiotherapy and radiotherapy, respectively. It is also important to note that endoscopic intervention may also be required to address complications of PRT (ie strictures) rather than address ongoing malignant obstruction^26^ although this is expected to be unlikely in the palliative setting. We report that approximately 5% of patients who received PRT underwent endoscopic dilation with or without stent insertion after completion of PRT. Due to the nature of the procedure codes we could not clearly differentiate between dilation and stent as several codes incorporated both; however, in the palliative setting it is our observation that stent insertion may be more common. We captured patients undergoing this procedure after the last course of PRT and for patients who required multiple courses of PRT not all of the procedures were counted. This may account, in part, for the low number of patients (5%) in our cohort that required this intervention; however, we do note that 83% of patients in the cohort only had one course of PRT. Information pertaining to need for secondary intervention is important for healthcare providers when having discussions with patients regarding expectations and outcomes of PRT.

The main strength of our study is the reporting of population-based real-world patterns in utilization and outcomes of radiation for patients with incurable esophageal or gastric cancer. To the best of our knowledge a similar study has not been performed using a population-level dataset; however, our study has limitations. We tried to limit our cohort to those who received radiation for palliative intent by excluding those who had received curative-intent surgery and radiotherapy, but it is possible that some of these patients were included in our cohort due to inaccuracies in surgical procedure and radiotherapy intent coding; however, the sensitivity analysis suggests that using the palliative intent code in the ALR database does identify the intended patient cohort. Using health administrative datasets for research is subject to other sources of bias including missing data and changing eligibility over time which may apply to our study.^13^ Also, the number of patients receiving PRT for gastric cancer was high in our cohort; however, we suspect that some of these patients had gastroesophageal junction tumors for whom there is no specific ICD-10 code and likely these patients are classified under both esophageal (C15) and gastric (C16) cancer codes.

Quality of life considerations are particularly critical in this population due to limited survival and high symptom burden^27^ and should be part of any conversation pertaining to initiation of palliative interventions. Ontario's health administrative data does contain some patient reported outcomes; however, they are broad and do not provide information in regard to alleviation of local symptoms (ie dysphagia relief). In general, outcomes pertaining to quality of life are lacking in assessments of PRT for esophageal and gastric cancer and are a critical area for future study. Our study assumes that PRT was delivered to address a specific local symptom (pain, bleeding, dysphagia, obstruction); however, we are not able to determine which specific symptom was being addressed and therefore can make no comment on how effective PRT was for relief of those symptoms. While population-based studies are well positioned to comment on general practice patterns and trends, they are unable to obtain granular information on clinical decision-making, patient and family preferences regarding treatment, variations in treatment delivery, and reasons behind provider recommendations. Finally, we have studied a cohort of patients who received treatment in Ontario, Canada. It is possible that the results of this study may not be generalizable to other provinces within Canada or elsewhere in the world.

## Conclusions

We show that PRT remains an important treatment in advanced esophageal and gastric cancer and that only a minority of patients will require subsequent endoscopic dilation with or without stent insertion after completion of PRT suggesting that PRT provides meaningful symptom control in the real world. We report that there is significant variation in the delivery of PRT for esophageal and gastric cancer in Ontario, Canada and that there is increasing utilization of advanced radiotherapy techniques for this indication. As survival remains poor in this disease, it is imperative that healthcare providers continue to look for ways of delivering treatment that minimizes toxicity and maximizes outcomes that are most valuable to patients.

## Appendix

[Table table3-08258597211072946] [Table table4-08258597211072946] [Table table5-08258597211072946] [Table table6-08258597211072946]

**Table A1. table3-08258597211072946:** Included Morphology Codes from the International Classification of Diseases for Oncology.

Morphology code	Description
80003	M8000/3 Neoplasm, malignant
80103	M-8010/3 Carcinoma, NOS
81403	M-8140/3 Adenocarcinoma, NOS
81423	M-8142/3 Linitis plastica (C16._)
81433	M-8143/3 Superficial spreading adenocarcinoma
81443	M-8144/3 Adenocarcinoma, intestinal type (C16._)
81453	M-8145/3 Carcinoma, diffuse type (C16._)
82103	M-8210/3 Adenocarcinoma in adenomatous polyp
82553	M-8255/3 Adenocarcinoma with mixed subtypes
82613	M-8261/3 Adenocarcinoma in villous adenoma
82623	M-8262/3 Villous adenocarcinoma
82633	M-8263/3 Adenocarcinoma in tubulovillous adenoma
84803	M-8480/3 Mucinous adenocarcinoma
84813	M-8481/3 Mucin-producing adenocarcinoma
84903	M-8490/3 Signet ring cell carcinoma

**Table A2. table4-08258597211072946:** Canadian Classification of Health Interventions (CCI) Procedural Codes for Excision/Resection of Esophagus and Stomach.

**Esophagus**	1NA87BA, 1NA87DA, 1NA87DB, 1NA87DBXXF, 1NA87DBXXG, 1NA87DC, 1NA87DD, 1NA87EY, 1NA87EZ, 1NA87FA, 1NA87FAXXF, 1NA87FAXXG, 1NA87FB, 1NA87FC, 1NA87LB, 1NA87LBXXF, 1NA87LBXXG, 1NA87LD, 1NA87LE, 1NA87LP, 1NA87LQ, 1NA87QB, 1NA87QBXXF, 1NA87QBXXG, 1NA87QC, 1NA87QD, 1NA87QF, 1NA87QFXXF, 1NA87QFXXG, 1NA87QG, 1NA87QH, 1NA88DCXXG, 1NA88FCXXG, 1NA88LBXXF, 1NA88LBXXG, 1NA88QFXXF, 1NA88QFXXG, 1NA89DB, 1NA89DBXXF, 1NA89DBXXG, 1NA89FA, 1NA89FAXXF, 1NA89FAXXG, 1NA89LB, 1NA89LBXXF, 1NA89LBXXG, 1NA89QF, 1NA89QFXXF, 1NA89QFXXG, 1NA90LBXXF, 1NA90LBXXG, 1NA90QFXXF, 1NA90QFXXG, 1NA91DB, 1NA91DBXXF, 1NA91DBXXG, 1NA91FA, 1NA91FAXXF, 1NA91FAXXG, 1NA91LB, 1NA91LBXXF, 1NA91LBXXG, 1NA91QF, 1NA91QFXXF, 1NA91QFXXG, 1NA92LBXXF, 1NA92LBXXG, 1NA92QFXXF, 1NA92QFXXG
**Stomach**	1NF87BA, 1NF87DA, 1NF87DG, 1NF87DH, 1NF87DJ, 1NF87DL, 1NF87DQ, 1NF87GX, 1NF87LA, 1NF87RG, 1NF87RH, 1NF87RJ, 1NF87RK, 1NF87RP, 1NF87SH, 1NF89DAXXF, 1NF89DZ, 1NF89GW, 1NF89LAXXF, 1NF89SG, 1NF89TH, 1NF90LAXXG, 1NF91LAXXF, 1NF91RG, 1NF91RJ, 1NF91RJXXF, 1NF91RP, 1NF91SG, 1NF92LAXXG

**Table A3. table5-08258597211072946:** Canadian Classification of Health Interventions (CCI) Procedural Codes for Esophageal Dilation with/Without Stent Insertion and Stomach Dilation.

Esophagus Dilation + /- stent insertion	1NA50BABD, 1NA50BABJ, 1NA50BABL, 1NA50BABP, 1NA50BANR, 1NA50BTBD, 1NA50BTBJ, 1NA50BTBL, 1NA50BTBP, 1NA50BTNR, 1NA50CABJ, 1NA50CRBJ
Stomach Dilation	1NF50BABL, 1NF50BABP

**Table A4. table6-08258597211072946:** Results of Sensitivity Analysis. Characteristics and Survival from Start of Palliative Radiation of Patients Identified as Having Received Palliative Radiotherapy by 1) Intent Code in ALR, 2) Dose/Fraction ≥300 cGy and 3) Dose/Fraction ≥250 cGy and <850 cGy and ≤ 15 Fractions.

	ALR intent (*n* = 2500)	Dose/fraction ≥ 300 cGy (*n* = 2511)	Dose/fraction ≥250 cGy and <850 cGy and ≤ 15 fractions (*n* = 2573)
**Age**			
≤40	50 (2.0%)	48 (1.9%)	52 (2.0%)
41 to 50	166 (6.6%)	182 (7.2%)	179 (7.0%)
51 to 60	442 (17.7%)	475 (18.9%)	479 (18.6%)
61 to 70	608 (24.3%)	618 (24.6%)	633 (24.6%)
≥71	1234 (49.4%)	1188 (47.3%)	1230 (47.8%)
mean (SD)	69.5 (13.4)	68.9 (13.3)	69.1 (13.4)
median (IQR)	70 (60-80)	69 (59-80)	70 (59-80)
**Sex**			
Male	1873 (74.9%)	1893 (75.4%)	1938 (75.3%)
Female	627 (25.1%)	618 (24.6%)	635 (24.7%)
**Charlson comorbidity index**			
0	927 (56.4%)	932 (57.0%)	949 (57.3%)
1	385 (23.4%)	368 (22.5%)	370 (22.3%)
≥ 2	332 (20.2%)	336 (20.5%)	337 (20.4%)
**Patient location**			
Rural	413 (16.6%)	428 (17.1%)	431 (16.9%)
Urban	2075 (83.4%)	2069 (82.9%)	2125 (83.1%)
**SES Quintile**			
1 (lowest)	448 (18.0%)	453 (18.1%)	466 (18.2%)
2	572 (23.0%)	563 (22.5%)	580 (22.6%)
3	572 (23.0%)	589 (23.5%)	601 (23.4%)
4	493 (19.8%)	502 (20.1%)	508 (19.8%)
5 (highest)	405 (16.3%)	395 (15.8%)	409 (16.0%)
**Distance to Nearest Hospital**			
≤ 10 kms	933 (37.5%)	948 (37.9%)	967 (37.7%)
11 to 50 kms	998 (40.1%)	993 (39.7%)	1024 (39.9%)
> 50 kms	559 (22.4%)	561 (22.4%)	573 (22.3%)
**Cancer site**			
Esophageal (C15.X)	1047 (41.9%)	1074 (42.8%)	1112 (43.2%)
Gastric (C16.X)	1453 (58.1%)	1437 (57.2%)	1461 (56.8%)
**Time from diagnosis to start of PRT**			
< 3 months	1881 (75.2%)	1955 (77.9%)	2013 (78.2%)
3 to 6 months	244 (9.8%)	216 (8.6%)	222 (8.6%)
6 to 12 months	229 (9.2%)	209 (8.3%)	206 (8.0%)
12 to 24 months	113 (4.5%)	102 (4.1%)	103 (4.0%)
24 months or more	33 (1.3%)	29 (1.2%)	29 (1.1%)
**Median Overall Survival (Days)**	108	106	110
**Median Cancer-Specific Survival (Days)**	110	108	112

ALR – Activity Level Reporting.
